# Targeting Autophagy Augments Berberine-Mediated Cell Death in Human Hepatoma Cells Harboring Hepatitis C Virus RNA

**DOI:** 10.3390/cells9040908

**Published:** 2020-04-08

**Authors:** Chen-Jei Tai, Alagie Jassey, Ching-Hsuan Liu, Cheng-Jeng Tai, Christopher D. Richardson, Shu Hui Wong, Liang-Tzung Lin

**Affiliations:** 1 Department of Traditional Chinese Medicine, Taipei Medical University Hospital, Taipei 110, Taiwan; chenjtai@tmu.edu.tw; 2 Department of Obstetrics and Gynecology, School of Medicine, College of Medicine, Taipei Medical University, Taipei 110, Taiwan; 3 International Ph.D. Program in Medicine, College of Medicine, Taipei Medical University, Taipei 110, Taiwan; d142105007@tmu.edu.tw; 4 Graduate Institute of Medical Sciences, College of Medicine, Taipei Medical University, Taipei 110, Taiwan; d119107007@tmu.edu.tw; 5 Department of Microbiology & Immunology, Dalhousie University, Halifax, NS B3H 4R2, Canada; chris.richardson@dal.ca; 6 Division of Hematology and Oncology, Department of Internal Medicine, Taipei Medical University Hospital, Taipei 110, Taiwan; cjtai@tmu.edu.tw; 7 Department of Internal Medicine, School of Medicine, College of Medicine, Taipei Medical University, Taipei 110, Taiwan; 8 Department of Pediatrics and Canadian Center for Vaccinology, Izaak Walton Killam Health Centre, Halifax, NS B3K 6R8, Canada; 9 International Master Program in Medicine, College of Medicine, Taipei Medical University, Taipei 110, Taiwan; m142107001@tmu.edu.tw; 10 Institut de Recherches Cliniques de Montréal, Montreal, QC H2W 1R7, Canada; 11 Department of Microbiology and Immunology, School of Medicine, College of Medicine, Taipei Medical University, Taipei 110, Taiwan

**Keywords:** autophagy, berberine, biphasic cell death, ROS

## Abstract

Hepatocellular carcinoma (HCC), including hepatitis C virus (HCV)-induced HCC, is a deadly disease highly refractory to chemotherapy, thus requiring the continuous identification of novel treatment strategies. Berberine (BBR) has been previously reported to inhibit hepatoma cell growth, but the main type of cell death elicited by BBR, and whether the alkaloid can inhibit hepatoma cells carrying HCV genomes, is unclear. Herein, we show that BBR treatment induced a biphasic cell death irrespective of the presence of HCV subgenomic replicon RNA, first triggering apoptosis that then progressed to necrosis between 24 and 48 h post-treatment. Furthermore, BBR treatment potentiated the HCV replicon-induced reactive oxygen species (ROS) production, inhibition of which with an antioxidant attenuated the cell death that was elicited by BBR in these cells. Moreover, BBR dampened the autophagic response in HCV RNA-positive or negative hepatoma cells, and pharmacological inhibition of autophagy conversely augmented the BBR-induced cell death. Finally, BBR inhibited the growth of Huh-7 cells that were persistently infected with the full-length genome HCV particles, and concomitant pharmacological inhibition of autophagy potentiated the killing of these cells by BBR. Our findings suggest that combining BBR with the inhibition of autophagy could be an attractive treatment strategy against HCC, irrespective of the presence of the HCV genome.

## 1. Introduction

The incidence of hepatocellular carcinoma (HCC), which is the most common cause of primary liver cancer, is on the rise. HCC is the sixth most common form of cancer and the second leading cause of cancer-related deaths globally [1]. Although the etiology of HCC is multifactorial, viral hepatitis due to hepatitis C virus (HCV) infection is an important predisposing factor, which causes over 20% of all HCC cases in both developed and developing countries [2].

Although the current treatment strategies, including orthotropic liver transplantation, surgical resection, and ablations, are effective against HCC at early stages, most cases detected at the advanced stages are unsuitable for such therapies [3]. Therefore, advanced cases are subjected to targeted therapies, such as sorafenib treatment [4]. However, the response rates remain poor, partially due to the genetic heterogeneity of HCC [5]. Moreover, HCC at the advanced stage displays poor prognosis with a survival time of fewer than six months in the untreated patients [6]. Although the advent of the direct-acting antivirals (DAAs) has phenomenally improved the treatment of HCV infection, which is a leading cause of HCC, these drugs do not reduce the development of HCV-associated HCC in all patients [7,8]. In fact, the use of the DAAs has been associated with the high occurrence of HCC in some settings [9]. Given that no effective vaccine still exists for the prevention of HCV infection, identifying/validating effective treatment strategies against HCC, particularly HCV-induced HCC, is urgently needed.

Plant-derived compounds are an important source of medicine and they have been extensively explored to treat a variety of diseases [10]. Berberine (BBR), a plant alkaloid and a quaternary ammonium salt that is widely found in traditional herbal medicines, is typically used to treat conditions such as diabetes and diarrhea [11]. The alkaloid has been reported to possess anti-cancer activities both in vitro and in vivo, including against hepatoma cells [12]. Specifically, BBR has been demonstrated to exert its anti-hepatoma activity by inducing apoptotic, necrotic, and autophagic cell deaths in various cell types [13,14,15]. However, precisely how BBR modulates these types of cell death and the main form of cell death that is elicited by BBR in the hepatoma cells in the context of HCV infection remains unclear.

Autophagy, which is also called type II cell death, is a stress response pathway that is evolutionarily conserved in eukaryotes and it plays a crucial role in maintaining cellular homeostasis. The process begins with the formation of a membrane crescent structure—phagophore/isolation membrane that surrounds the cargo targeted for degradation [16]. The phagophore subsequently elongates via two ubiquitin-like conjugation processes—lipidation of the microtubule-associated protein 1 light chain 3 (LC3I) to form LC3II, and the conjugation of the autophagy-related proteins (ATG5 to ATG12 and ATG16L), to form a double membrane structure that is known as the autophagosome. The autophagosome matures by fusing with lysosomes, which leads to the degradation of the cargo and the eventual recycling of amino acids for biogenesis [17]. Although autophagy can also act as an antimicrobial defense mechanism through a process called xenophagy, several RNA viruses have been documented to usurp the autophagy pathway for their benefit [18]. HCV is one such virus that induces autophagy to prevent apoptosis, promote its replication, and, at the same time, inhibit the innate antiviral immune response [19].

Herein, we investigated the effect of BBR treatment on hepatoma cells with or without the presence of intracellular HCV RNA replication, given the importance of HCV infection in the development of HCC, and to determine the main mechanism of hepatoma cell death that is induced by BBR in the context of HCV infection. Our results demonstrated that BBR treatment induces a time-dependent biphasic cell death in both the Huh-7 cells and Huh-7 replicon cells carrying subgenomic HCV RNA. Moreover, BBR treatment enhanced reactive oxygen species (ROS) production in HCV replicon cells without exerting any significant impact on the HCV-negative parental Huh-7 cells. Consequently, the inhibition of ROS with an antioxidant only attenuated the BBR-induced cell death in the HCV subgenomic replicon cells and not in the parental Huh-7 cells. Furthermore, BBR treatment robustly attenuated autophagy in both cell types, as demonstrated by a decrease in both LC3I and LC3II in the presence of the natural alkaloid, and the pharmacological inhibition of autophagy with bafilomycin A1 (BAF) augmented the BBR-induced cell death. Finally, we show that BBR could equally inhibit the growth of Huh-7 cells persistently harboring full-length HCV genomes by similarly modulating autophagy, and that concomitant BAF treatment potentiated the killing of these cells by BBR. Our results demonstrate that BBR can inhibit hepatoma cell growth, irrespective of intracellular HCV RNA expression, and further suggest that combining BBR with an autophagy inhibitor might enhance the treatment efficacy of this alkaloid against HCC. These outcomes warrant further investigation on developing molecular therapies that are based on such strategies for the management of HCC.

## 2. Results

### 2.1. BBR Inhibits Hepatoma Cell Growth Irrespective of HCV RNA Expression

**Figure 1:**
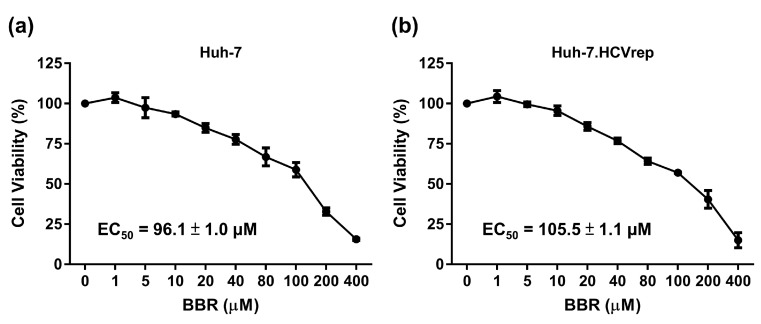
Berberine (BBR) inhibited the viability of hepatoma cells. (**a**) Huh-7 and (**b**) Huh-7.HCVrep cells were seeded in 96-well plates (2 × 10^4^ cells/well) and treated with the indicated concentrations of BBR for 72 h before cell viability was analyzed with Cell Counting Kit-8 (CCK-8). The results are shown as means ± SD from three independent repeats. The EC_50_ values are shown in the respective graphs.

BBR is a plant alkaloid that has been demonstrated to possess anti-tumor effects against a variety of cancer cell lines, including hepatoma cells [20]. However, the exact mechanism of the BBR-induced hepatoma cell death, and whether or not the small molecule possesses anti-tumor activities in the context of HCV infection remains unclear. We asked whether BBR could inhibit the growth of the HCV RNA-containing hepatoma cells, given the importance of HCV infection in inducing HCC. To this end, Huh-7 and the HCV replicon Huh-7.HCVrep cells (Huh-7 cells expressing the HCV genotype 1 subgenomes) were treated with various concentrations of BBR (0, 1, 5, 10, 20, 40, 80, 100, 200, and 400 μM) for 72 h, followed by CCK-8 cell viability analysis. BBR treatment dose-dependently inhibited the proliferation of both the naïve Huh-7 cells and the Huh-7.HCVrep replicon cells, as depicted in Figure 1a,b. The EC_50_ values were comparable (Huh-7: 96.1 ± 1.0 μM vs. Huh-7.HCVrep: 105.5 ± 1.1 μM). These results indicated that the alkaloid could inhibit hepatoma cell growth, irrespective of HCV RNA expression.

### 2.2. BBR Induces Time-Dependent Biphasic Hepatoma Cell Death

**Figure 2:**
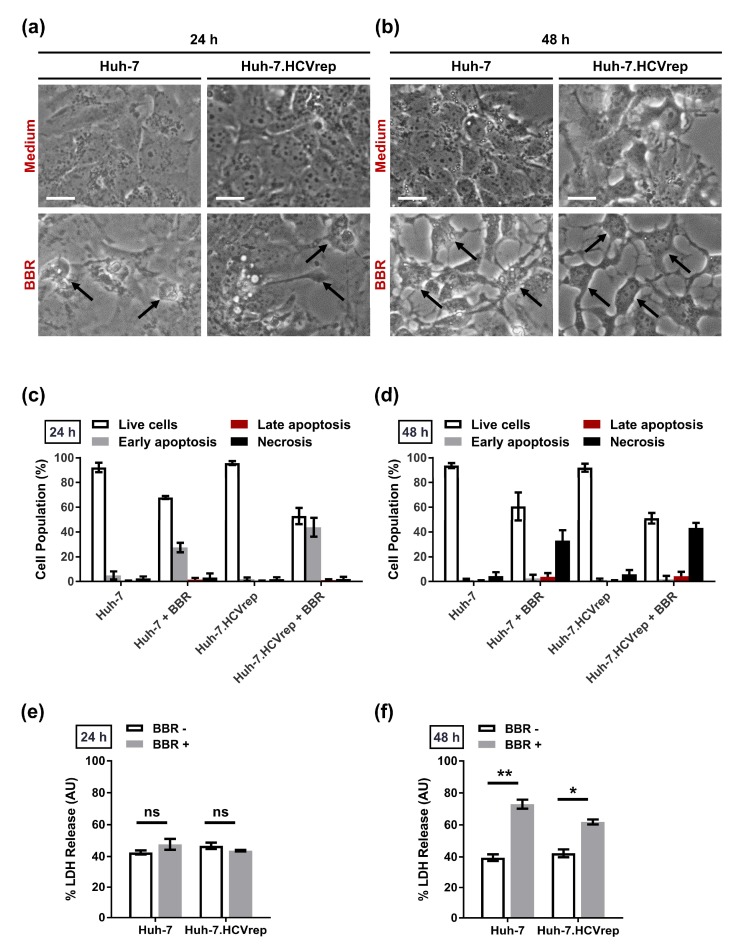
Berberine (BBR) induced biphasic cell death in the hepatoma cells. (**a,b**) Huh-7 and Huh-7.HCVrep cells were seeded in six-well plates (5 × 10^5^ cells/well) and treated with or without 100 µM BBR for (**a**) 24 or (**b**) 48 h. Changes in the morphology of the cells were observed using a microscope (indicated by black arrows). Magnification = 200×; scale bar = 25 μm. For cell death analysis, cells treated for (**c**) 24 or (**d**) 48 h were subjected to Annexin V/Propidium Iodide (PI) staining for flow cytometry cell death analysis. For lactose dehydrogenase (LDH) release assay, cells were seeded in 96-well plates and treated with 100 µM BBR for (**e**) 24 or (**f**) 48 h before analysis of LDH release. Results are shown as means ± SD from three independent repeats for a, b, c, and d, and two independent repeats for e and f. * *p* < 0.05; ** *p* < 0.01; ns = not significant.

Huh-7 and the Huh-7.HCVrep cells were treated with 100 μM of BBR for 24 or 48 h before being subjected to Annexin V/Propidium Iodide (PI) flow cytometric analysis to better understand how BBR inhibits hepatoma cell proliferation. We chose 100 μM of BBR for all subsequent experiments, unless otherwise stated, because this concentration does not induce massive cell death at the timepoints tested, thus allowing for us to determine the underlying mode of cell death that is caused by BBR treatment. Prior to Annexin V/PI staining, the cells were microscopically examined for morphological changes induced by the drug treatment. in contrast to the untreated controls, BBR treatment induced alterations in the morphology of the cells, particularly at 48 h post-treatment, regardless of the presence of HCV RNA, as demonstrated in Figure 2a,b. The Annexin V/PI flow cytometry analysis showed that BBR treatment mainly induced early apoptotic cell death (increased Annexin V-positive/PI-negative population) at 24 h post-treatment (Figure 2c). In contrast, at 48 h post-treatment, the form of cell death shifted to predominantly necrosis, as indicated by the increase in the PI only-positive cell populations (Figure 2d). We conducted a lactose dehydrogenase (LDH) release assay, which is known to be increased during necrotic cell death, to further support the induction of biphasic cell death by BBR [21]. LDH is a cytosolic enzyme that is released in the extracellular space when the plasma membrane is disrupted and, hence, is commonly used as a marker for necrosis [22]. Whereas no significant difference in LDH release in the presence or absence of BBR was observed at 24 h post-treatment in both HCV RNA-positive and negative hepatoma cells (Figure 2e), the compound significantly induced LDH release at 48 h post-treatment as compared to the controls (Figure 2f). These results suggested that BBR treatment induced a time-dependent biphasic hepatoma cell death.

### 2.3. Inhibition of ROS Attenuates the BBR-Induced HCV Replicon Cell Death, But Not the Parental HCV RNA-Negative Huh-7 Cells

**Figure 3:**
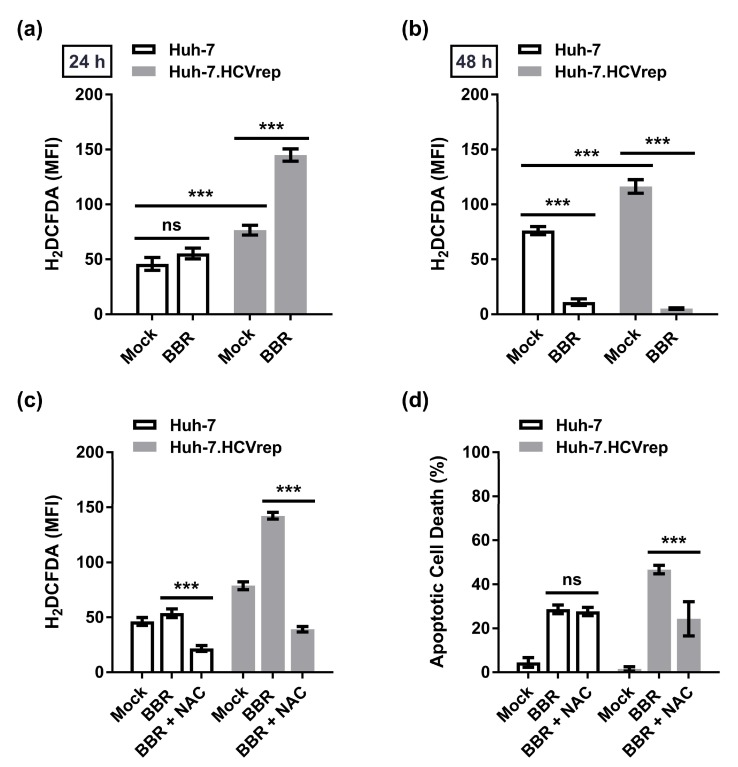
N-acetyl-cysteine (NAC) attenuated berberine (BBR)-induced cell death in the Huh-7 cells carrying hepatitis C virus (HCV) subgenomic replicon RNA. Huh-7 and Huh-7.HCVrep cells were seeded in 6-well plates and treated with or without 100 μM BBR for (**a**) 24 or (**b**) 48 h. Cells were then stained with 20 μM 2’,7’-dichlorodihydrofluorescein diacetate (H_2_DCFDA) for flow cytometry analysis. For reactive oxygen species (ROS) inhibition analysis, cells seeded in 6-well plates were pretreated with 10 mM NAC for 48 h before treatment with 100 μM BBR for 24 h and subjected to (**c**) H_2_DCFDA staining or (**d**) Annexin V/Propidium Iodide (PI) staining. Results are shown as means ± SD from three independent repeats for all experiments. *** *p* < 0.001; ns = not significant.

The above results demonstrated that BBR induced biphasic cell death—first triggering apoptosis that then progressing to necrotic cell death at 48 h post-treatment. Next, we sought to investigate the underlying mechanism(s) of the BBR-induced cell death. Given the importance of ROS in regulating many biological processes, including cell death [23], we examined whether BBR treatment could modulate ROS production in the hepatoma cells. The HCV replicon Huh-7.HCVrep cells and the HCV RNA-negative parental Huh-7 cells were treated with or without BBR for 24 or 48 h before staining with H_2_DCFDA dye, an indicator of ROS formation [24], and analyzed by flow cytometry. Although BBR treatment only marginally increased ROS production in Huh-7 cells at 24 h post-treatment, the drug robustly upregulated the Huh-7.HCVrep-induced ROS production, as indicated in Figure 3a. Analysis of ROS at 48 h showed a significant decrease in the ROS levels in the treatment groups when compared to the mock control for both cells, which we attribute to the increase in the BBR-induced cell death at this timepoint (Figure 3b). Next, we asked whether N-Acetyl Cysteine (NAC) treatment, a well-known antioxidant and inhibitor of ROS [25], could inhibit the BBR-mediated induction of ROS in these cells. The cells were pretreated for 48 h with NAC and subsequently treated with BBR for 24 h before performing H_2_DCFDA staining analysis. Indeed, NAC pretreatment abrogated ROS production in both BBR-treated HCV replicon cells and parental Huh-7 cells to below basal levels, as demonstrated in Figure 3c. We then investigated whether the inhibition of ROS using NAC could impact BBR-induced cell death while using the same treatment method but analyzed through Annexin V/PI staining. While inhibition the of ROS had no significant effect on the BBR-induced apoptotic cell death in Huh-7 cells, likely due to the absence of significant ROS induction, NAC pretreatment significantly inhibited the BBR-mediated apoptotic cell death in the Huh-7.HCVrep cells, as depicted in Figure 3d. These results suggested that ROS plays an important role in the BBR-mediated apoptotic cell death of the Huh-7.HCVrep cells, but not in the parental HCV RNA-negative Huh-7 cells.

### 2.4. BBR Modulates Autophagy in HCC Cells

**Figure 4:**
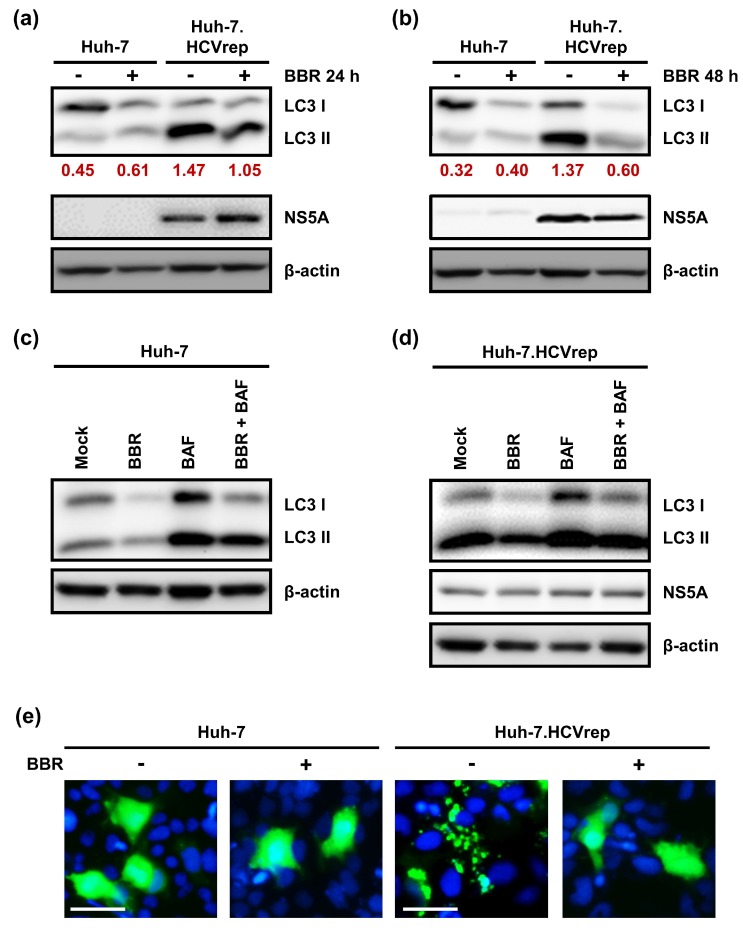
Berberine (BBR) modulated autophagy in the hepatoma cells. Huh-7 and Huh-7.HCVrep cells were seeded in six-well plates and treated with or without 100 µM BBR for (**a**) 24 or (**b**) 48 h. The lysates were collected at the end of the incubation periods for a Western blot analysis against microtubule-associated protein 1 light chain 3 (LC3). Densitometry was calculated by normalization of LC3II expressions to β-actin. (**c**) Huh-7 and (**d**) Huh-7.HCVrep cells seeded in six-well plates were treated either with BBR alone, bafilomycin A1 (BAF) alone, or pretreated with BAF for 4 h before treating with BBR for an additional 24 h. The whole cell lysates were collected and analyzed by Western blotting. For all Western blot analyses, nonstructural protein 5A (NS5A) expression indicated the presence of hepatitis C virus (HCV) replicon, and β-actin was used as a loading control. (**e**) Huh-7 and Huh-7.HCVrep cells that were seeded in 24-well plates were transfected with the enhanced green fluorescent protein (EGFP)-LC3 plasmid overnight and treated with or without BBR for 48 h. The cells were then fixed, stained with the Hoechst nuclear stain, and then viewed using a fluorescence microscope. Magnification = 200×; scale bar = 40 μm.

Autophagy is a lysosome-dependent catabolic pathway that is implicated in promoting cell survival under stressful conditions [17]. HCV is known to upregulate autophagy to maintain cell survival and, hence, promote persistent viral replication [26,27,28]. On the other hand, hepatocytes are known to induce autophagy at the basal level to maintain cellular homeostasis [29]. When considering the importance of autophagy in maintaining cell survival in the hepatoma cells, we next asked whether BBR treatment could alter autophagy in these cells. Huh-7 and the Huh-7.HCVrep cells were treated with or without BBR for 24 or 48 h before Western blot was performed in order to analyze the autophagy marker, LC3. LC3 exists in two forms; the cytosolic or non-lipidated form LC3I, which is covalently linked to phosphatidylethanolamine to generate the lipidated form LC3II upon the induction of autophagy. The conversation of LC3I to LC3II is the hallmark of autophagy [30]. The results in Figure 4a,b demonstrated that, while the parental Huh-7 cells did not induce LC3II formation at both timepoints, the Huh-7.HCVrep cells robustly induce autophagy, as indicated by the high level of LC3II expression in these cells, indicating that HCV replication triggers the autophagic response. Interestingly, we observed that BBR treatment inhibited both the LC3I and LC3II expressions, particularly at 48 h post-treatment in both cells. The decrease in LC3 expression in the presence of BBR could suggest either the inhibition or potentiation of the autophagic process. The cells were prepared either untreated, treated with BBR or the autophagy inhibitor bafilomycin (BAF) alone, or pretreated with BAF before adding BBR for Western blot analysis, in order to understand how BBR modulates autophagy. The results in Figure 4c,d indicated that while BAF treatment alone resulted in the accumulation of LC3II, indicating the efficient induction of autophagic flux, BAF pretreatment followed by BBR treatment did not further increase the BAF-induced LC3II accumulation. These results suggest that the decrease in LC3 lipidation that was observed after BBR treatment was due to the inhibition of autophagy, rather than its potentiation. We performed an EGFP-LC3 immunofluorescence assay to monitor the LC3-associated punctate formation, which is increased during autophagy, to validate the effect of BBR treatment in impeding autophagy in these cells. For this purpose, the cells were transfected with the EGFP-LC3 plasmid and then treated with or without BBR for 24 h for immunofluorescence microscopy. In contrast to the parental Huh-7 cells, which showed a diffused pattern (no autophagy induction) of EGFP-LC3, with or without the presence of BBR, the Huh-7.HCVrep cells had substantially induced punctate formation, and treatment with BBR reversed this phenomenon (Figure 4e). Together, these results suggest that BBR inhibits autophagy in the hepatoma cells.

### 2.5. BAF Pretreatment Augments BBR-Induced Cell Death in Hepatoma Cells Irrespective of Intracellular HCV RNA Replication 

**Figure 5:**
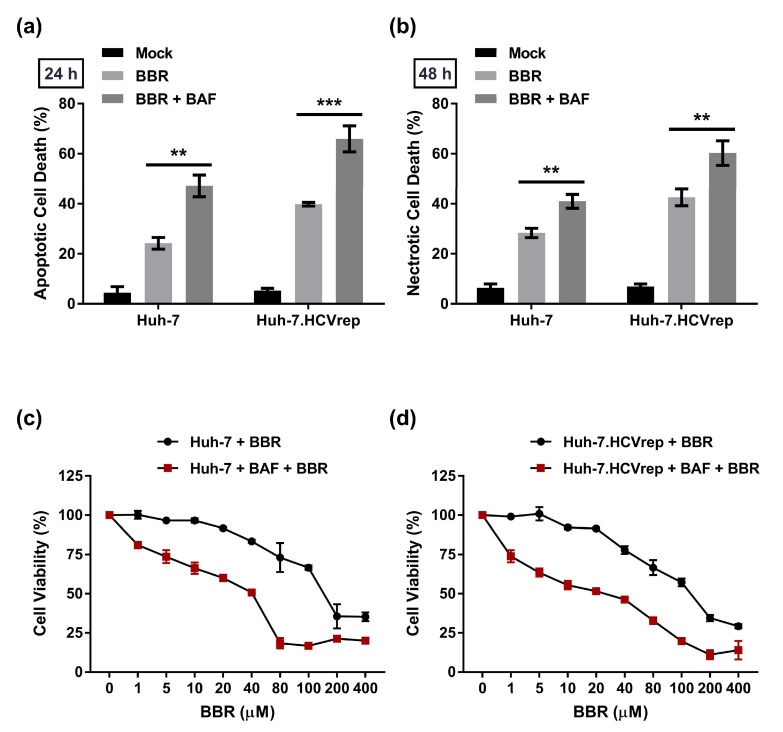
Bafilomycin A1 (BAF) pretreatment augmented BBR-induced cell death in the hepatoma cells. Huh-7 and Huh-7.HCVrep cells were seeded in six-well plates and pretreated with 100 nM BAF for 4 h. The cells were then treated with 100 µM berberine (BBR) for (**a**) 24 or (**b**) 48 h before Annexin V/Propidium Iodide (PI) staining for flow cytometry analysis. (**c**) Huh-7 and (**d**) Huh-7.HCVrep cells were seeded in 96-well plates and pretreated with BAF, as above. The cells were subsequently treated with BBR at the indicated concentrations for 48 h before assessment using Cell Counting Kit-8 (CCK-8) cell viability analysis. The results are shown as means ± SD from 3 independent repeats for a and b, and two independent experiments for c and d. ** *p* < 0.01; *** *p* < 0.001.

Following the above observation that BBR inhibited autophagy in the hepatoma cells, we next sought to investigate the relevance of autophagy inhibition in the BBR-mediated cell death. To this end, we pretreated the cells with the autophagy inhibitor BAF for 4 h, followed by BBR treatment for 24 or 48 h prior to Annexin V/PI flow cytometry analysis. The results show that BAF pretreatment enhanced the BBR-induced cell death at both timepoints (triggering apoptosis at 24 h and leading to necrosis at 48 h) in both Huh-7 cell types with or without intracellular HCV RNA replication (Figure 5a,b). We pretreated cells with BAF for 4 h, followed by BBR treatment for 48 h before assessing cell viability using CCK-8 analysis, to confirm that inhibition of autophagy through BAF treatment enhances the BBR-induced cell death. The results in Figure 5c demonstrated that BAF pretreatment markedly reduced the EC_50_ value of Huh-7 cells that were treated with BBR alone for 48 h from 162.8 ± 0.4 μM to 23.0 ± 0.1 μM. Similar results were observed in the Huh-7.HCVrep cells, wherein BAF pretreatment reduced the EC_50_ value from 130.7 ± 0.5 μM to 19.7 ± 0.7 μM (Figure 5d). These results, in combination with the above observations, suggested that BBR inhibits autophagy to induce hepatoma cell death. Interestingly, similar to our findings, the inhibition of autophagy was previously reported to enhance necrotic cell death [31]. Finally, in support of the notion that BAF treatment augments the BBR-induced cell death, we demonstrated that co-treatment of the hepatoma cells with low concentration of both agents (5 nM BAF and 5 μM BBR) for 48 h significantly induced the hepatoma cell death to a similar degree to BBR alone at 100 μM (Figure S1), which suggested the anti-HCC potency of the combination treatment.

### 2.6. Inhibition of Autophagy Enhances BBR-Induced Death of Cells Persistently Infected With HCV Particles

**Figure 6:**
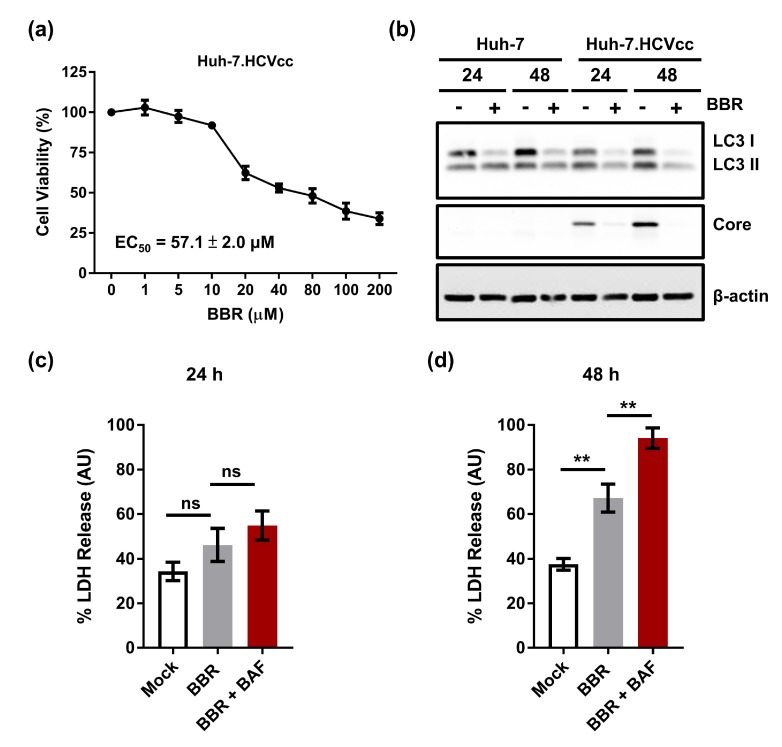
The inhibition of autophagy enhances berberine (BBR)-induced death of cells persistently infected with hepatitis C virus (HCV) particles. (**a**) Huh-7.HCVcc cells were seeded in 96-well plates (2 × 10^4^ cells/well) overnight and treated with the indicated concentrations of BBR for 72 h before cell viability analysis. (**b**) Huh-7 and Huh-7.HCVcc cells were seeded in six-well plates and subsequently treated with 100 µM BBR for 24 or 48 h. Lysates where then collected and subjected to Western blot analysis against microtubule-associated protein 1 light chain 3 (LC3). HCV core expression indicated the presence of infection, and β-actin was used as a loading control. For cell death analysis, cells were seeded in 96-well plates and either left untreated, treated with 100 µM BBR alone for (**c**) 24 or (**d**) 48 h, or pretreated with bafilomycin A1 (BAF) for 4 h, followed by BBR treatment for 24 or 48 h before lactose dehydrogenase (LDH) release analysis. Results are shown as means ± SD from three independent repeats for all experiments. ** *p* < 0.01; ns = not significant.

Finally, we examined the effect of the plant alkaloid treatment on the viability of Huh-7 cells persistently infected with the cell culture-derived full-length genome HCV particles to further confirm the effect of BBR in inhibiting hepatoma cells expressing the HCV RNA (Huh-7.HCVcc cells). Our cytotoxicity analysis showed that BBR treatment robustly inhibited the Huh-7.HCVcc cell growth, with an EC_50_ value of 57.1 ± 2.0 μM (Figure 6a), which is nearly two-fold lower than those that were observed for both the parental Huh-7 cells and the HCV replicon Huh-7.HCVrep cells and, therefore, highlights the efficacy of BBR in inhibiting cells that were infected by HCV particles. Given our results that BBR modulates autophagy in the Huh-7 and Huh-7.HCVrep cells, we next asked whether BBR could exert a similar effect on the Huh-7.HCVcc cells. To this end, the cells were treated with or without BBR for Western blot analysis against the indicated autophagy proteins. Figure 6b shows that similar to the Huh-7 and the Huh-7.HCVrep cells, BBR treatment inhibited the Huh-7.HCVcc-induced LC3 lipidation, particularly at 48 h post-treatment, which suggests that the drug inhibits autophagy in these cells. Finally, the cells were either left untreated or pretreated with BAF before the addition of BBR for LDH assay to examine whether inhibition of autophagy using BAF could equally modulate the BBR-induced Huh-7.HCVcc necrotic cell death. When compared to the mock control group at 24 h, BBR treatment only marginally increased the LDH release, with BAF pretreatment showing a negligible effect on the BBR-induced LDH release, as shown in Figure 6c. In contrast, at 48 h, BBR treatment remarkably increased the LDH release when compared to the mock control, which was augmented by BAF pretreatment (Figure 6d), suggesting that BBR also induces time-dependent necrotic cell death in the Huh-7.HCVcc cells. Taken together, our results demonstrated that BBR could inhibit the hepatoma cell growth with or without infectious HCV RNA replication, and that combining BBR with an autophagy inhibitor, such as BAF, could be an attractive strategy for the treatment of HCC.

## 3. Discussion

HCC is highly refractive to treatment and it is a leading cause of cancer-related deaths worldwide [1]. Thus identifying/validating efficient and effective treatment strategies against this malignant disease is crucial. Nature-derived compounds are an important source of drug discovery against various conditions, including HCC. Recently, several natural compounds have been discovered to possess potent anti-HCC activity, including curcumin and its analog, EF25-(GSH)2 [32,33], resveratrol [34], tanshinone IIA [35], and silibinin [36]. Our findings that BBR could inhibit hepatoma cell growth, further strengthening the body of evidence on the anti-HCC bioactivity of this alkaloid [13,14,15], and adds it to the growing list of natural products as lead compounds that warrant further evaluation for the development as anti-HCC therapeutics.

BBR was previously reported to induce apoptotic cell death in the Huh-7 human hepatoma cells at various timepoints (24, 48, and 72 h) post-treatment [14]. Partly in agreement with this report, we also observed the induction of apoptosis at 24 h post-BBR treatment (Figure 2c). However, unlike the above study, we saw a switch in the form of cell death from apoptosis to predominantly necrosis at 48 h post-treatment (Figure 2d). In support of the induction of necrotic cell death at 48 h post-treatment, the LDH assay, which is a well-known indicator for necrotic cell death [21], showed a negligible difference between the mock control groups (Huh-7 and Huh-7.HCVrep cells only) and the BBR-treated groups at 24 h post-treatment (Figure 2e), whereas BBR treatment for 48 h significantly increased LDH release (Figure 2f). Moreover, the analysis of poly (ADP-ribose) polymerase (PARP) cleavage, a well-described indicator of apoptotic cell death [37], showed no significant difference between the mock control groups and the BBR-treated groups at both timepoints (Figure S2), suggesting that BBR mainly induces necrotic cell death at the late timepoint. It is possible that the drug could modulate cell death in a time/concentration-dependent manner, with higher concentrations inducing necrotic cell death, given that the authors of the aforementioned study used 10–20 µM of BBR in their study vs. 100 µM in our study. In support of this notion, Zhang et al. demonstrated that HepG2 cells that were treated with 100 µM of BBR elicited both apoptotic and necrotic death phenotypes [15]. How exactly BBR induces biphasic cell death in the hepatoma cells is unclear. Previous studies have shown that the cellular adenosine triphosphate (ATP) level is a key determinant in the switch between apoptotic and necrotic cell deaths, with high ATP levels favoring apoptotic cell death [38]. Given that BBR has been previously reported to decrease the ATP level in C2C12 myotubes [39], the alkaloid might deplete the ATP in hepatoma cells at the late timepoints to induce necrotic cell death. Further research would be needed in order to determine exactly how BBR modulates hepatoma cell death and clarify, for instance in our case, how the drug treatment time-dependently switches the form of cell death from apoptosis to necrosis.

HCV infection is known to induce oxidative stress in human hepatoma cell lines to favor persistent viral infection [40,41]. Consistent with the induction of oxidative stress by HCV, we showed that the Huh-7.HCVrep cells produced a higher level of ROS when compared to the parental Huh-7 cells (Figure 3a,b). More importantly, BBR treatment potentiated the replicon-induced ROS production, and the inhibition of ROS with NAC attenuated alkaloid-mediated death in these cells (Figure 3a,d). Unlike the Huh-7.HCVrep cells, BBR treatment did not modulate ROS production in the HCV-negative parental Huh-7 cells and, hence, the antioxidant treatment had no significant effect its BBR-induced cell death (Figure 3d). Of note, we observed that BBR appears to be fairly more effective in inducing apoptotic and necrotic cell death in the Huh-7.HCVrep cells than the naïve cells (Figure 2c,d). Interestingly, BBR has been shown to promote cancer cell death by potentiating ROS production [42], which is known to cause DNA damage [43]. Thus, the observation that the replicon cells exhibit moderately greater BBR-induced cell death than the naïve Huh-7 cells could be partly attributed to the drug-induced over-amplification of ROS production in these cells, leading to more cell death.

Our recent study demonstrated that BBR at low and non-cytotoxic concentration also impedes HCV infection of Huh-7 cells by targeting early viral entry events [44]. Specifically, we show that the alkaloid treatment could specifically block HCV particle attachment and entry/fusing into the host cells, thereby identifying the small molecule as a potent HCV entry inhibitor. The use of the compound could provide dual benefits in the context of HCV-induced HCC, given our results that BBR treatment at higher cytotoxic concentration also effectively inhibited the HCV RNA-containing Huh-7 replicon cell proliferation. For example, the drug treatment could attenuate hepatoma cell growth, while at the same time block viral infectivity of the host cells and restrict viral spread. Interestingly, we also observed that BBR at a non-cytotoxic concentration marginally blocked HCV replication as has been previously described (Figure S3) [44]. Combined, these results suggest that BBR could be further explored as an attractive candidate compound for the treatment of HCC in the context of HCV infection.

BBR was previously reported to cause autophagic cell death in HepG2 and MHCC97-L hepatoma cells [13]. Specifically, the authors demonstrated that BBR treatment concentration-dependently increased LC3II lipidation and that the inhibition of autophagy using 3-methyladenine (3-MA) attenuated BBR-induced cell death. In contrast, our results demonstrated that BBR treatment significantly inhibited autophagy, as indicated by the substantial loss of LC3II in the parental Huh-7 cells and the HCV expressing hepatoma cells (Huh-7.HCVrep and Huh-7.HCVcc), particularly at the 48 h post-treatment (Figure 4a,b and Figure 6b). Furthermore, pre-treatment using the autophagy inhibitor BAF potentiated BBR-mediated cell death (Figure 5 and Figure 6d), supporting the notion that the alkaloid blocks autophagy to cause hepatoma cell death. Our finding that BBR impedes autophagy to induce hepatoma cell death is therefore plausible, given the importance of autophagy in maintaining cell survival, including cancer cell survival in response to chemotherapy [43]. Interestingly, BBR was reported to attenuate autophagy in adipocytes by inhibiting beclin 1 expression [45], a key gene involved in the initiation of autophagy. Whether or not the drug employs a similar mechanism to inhibit autophagy in the hepatoma cells remains to be clarified. Nonetheless, our observations point to a potential strategy of using BBR in combination with an autophagy inhibitor to achieve enhanced hepatoma cell death.

In summary, we report here that BBR treatment induces biphasic cell death in the human hepatoma cells, irrespective of HCV RNA replication, and that the inhibition of autophagy using BAF increases BBR-induced cell death. Overall, our results suggest that combining BBR with autophagy inhibition represents an attractive treatment strategy against HCC, including in the context of HCV infection, and it merits further exploration in such a scenario. Further investigation of BBR treatment should also be carried out on normal hepatocytes, such as using the primary human hepatocytes model, to help determine the safety of BBR for its potential clinical development.

## 4. Materials and Methods

### 4.1. Chemicals and Reagents

Dimethyl sulfoxide (DMSO), N-acetyl-cysteine (NAC), and the autophagy inhibitor bafilomycin A1 (BAF) were purchased from Sigma–Aldrich (St. Louis, MO, USA). Phosphate buffered saline (PBS) was purchased from Thermo Fisher Scientific (Waltham, MA, USA). Fetal bovine serum (FBS), gentamycin, amphotericin B, and Dulbecco’s modified Eagle’s medium (DMEM) were purchased from Life Technologies (Carlsbad, CA, USA). The Annexin V-FITC Apoptosis Detection Kit was purchased from eBioscience (San Diego, CA, USA).

### 4.2. Cell Culture

The human hepatoma Huh-7 cells were cultured in DMEM containing 10% FBS, 1% gentamycin, 1% amphotericin B, and incubated at 37 °C in a 5% CO_2_ incubator. The AB12-A2 Huh-7 replicon cells carrying the HCV genotype 1b subgenomic RNA (denoted as ‘Huh-7.HCVrep’) were similarly maintained in DMEM, but supplemented with 1 mg/mL of G418 (InvivoGen; San Diego, CA, USA), as previously described [46]. Huh-7 cells persistently producing infectious cell culture-derived HCV particles (denoted as ‘Huh-7.HCVcc’) were initially established by electroporation of the full-length HCV genome Jc1FLAG2 (p7-nsGluc2A), as previously described [47], and then continuously passaged in media containing HCVcc particles until all cells tested positive for HCV NS5A expression. The Huh-7.HCVcc cells were maintained in medium as the parental Huh-7 cells.

### 4.3. Cytotoxicity Assay

The cells were seeded in 96-well plates (2 × 10^4^ cells/well) and then treated with increasing concentrations of BBR (0, 1, 5, 20, 40, 80, 100, 200, and 400 µM) for 72 h. Cell viability was analyzed using Cell Counting Kit-8 (CCK-8; Sigma), following the manufacturer’s instruction. The effective concentration of the drug that inhibited 50% of cell growth (EC_50_) was calculated while using GraphPad Prism software (V7.03, GraphPad Software; San Diego, CA, USA).

### 4.4. Analysis of Cell Death

Cell death due to BBR treatment was assayed using the Annexin V/Propidium Iodide (PI) apoptosis detection kit, as per the manufacturer’s instructions. Briefly, the cells were cultured in six-well plates (5 × 10^5^ cells/well) overnight and then treated with 100 μM of BBR for 24 or 48 h. The cells were afterward trypsinized, washed once with PBS, and then re-suspended in binding buffer. The Annexin V FITC (eBioscience) was then added to the cells and incubated at room temperature for 10 min. The cells were subsequently washed with the binding buffer (1X) and re-suspended in 190 μL of fresh binding buffer. Finally, 10 µL of PI was added to the cells and the samples were immediately analyzed while using the BD FACSCalibur flow cytometer and the associated CellQuest Pro software (BD Biosciences; San Jose, CA, USA).

### 4.5. Reactive Oxygen Species Production and Scavenging Analysis

Reactive oxygen species (ROS) production was analyzed using 2’,7’-dichlorodihydrofluorescein diacetate (H_2_DCFDA; Sigma). Briefly, the cells were seeded in six-well plates overnight and then treated with 100 µM of BBR for 24 or 48 h. For the ROS scavenging assay, cells were pre-treated with 10 mM NAC for 48 h before treating with 100 µM BBR for an additional 24 h. The cells were then stained with 20 μM H_2_DCFDA for 30 min at 37 °C, after which they were trypsinized, washed twice with PBS, and then re-suspended in ice-cold PBS for flow cytometry analysis, as described above.

### 4.6. Bafilomycin A1 Treatment

For assessing the effect of bafilomycin A1 (BAF) treatment on the BBR-mediated autophagy inhibition, the seeded cells in the respective experiments were either treated with (i) BBR alone, (ii) BAF alone, or (iii) pretreated with BAF for 4 h, followed by BBR treatment, and then incubated for an additional 24 or 48 h before the cells were prepared for the respective analyses.

### 4.7. Lactose Dehydrogenase Activity Assay for the Detection of Necrosis

The lactose dehydrogenase (LDH) assay was conducted, as previously described [21]. Briefly, the cells were seeded in 96-well plates and then treated with the various concentrations of the test compounds for 24 or 48 h. To generate the maximum LDH release control, 10 µL of the 10× lysis solution was added to the control wells and then incubated at 37 °C for 45 min. At the end of the incubation, 50 µL of the supernatants were transferred into a flat-bottom 96-well plate, to which 50 µL of the LDH substrate was added, mixed, and covered with aluminum foil and incubated at room temperature for 30 min. Finally, 50 µL of the stop solution was added and the absorbance was immediately read at 490 nm while using an ELISA plate reader. The % LDH release was calculated using the following formula: % LDH release = (corrected reading from test wells-corrected reading from untreated wells)/(corrected maximum LDH release control-corrected reading from untreated wells).

### 4.8. Western Blotting

Cells that were cultured in six-well plates in the presence or absence of BBR treatment were lysed with RIPA buffer (Sigma) that was supplemented with cOmplete^TM^ Tablets Mini Protease Inhibitor Cocktail (ROCHE; Basel, Switzerland) for 30 min on ice. The cells were then clarified at 12000 RPM for 30 min, followed by protein quantitation using the Bio-Rad Protein Assay Kit II (Bio-Rad Laboratories; Hercules, CA, USA). Afterward, the whole cell lysates were separated using SDS-PAGE, and then transferred to a PVDF membrane for probing with the following primary antibodies: rabbit anti-LC3 antibody (Thermo Fisher Scientific) at 1:1000; mouse anti-HCV NS5A (Millipore; MAB8694) at 1:250; mouse anti-HCV core (C7-50) (Thermo Fisher Scientific) at 1:400; and, mouse anti-β-actin (C4) (Santa Cruz Biotechnology; Santa Cruz, CA, USA) at 1:1000. The secondary antibodies that included goat anti-rabbit IgG H&L HRP (Abcam) and anti-mouse IgG HRP (Thermo Fisher Scientific) were used at a 1:3000 dilution. The membranes were finally overlaid with ECL (Bio-Rad) before acquiring the images while using the ChemiDoc-ItTS2 imager (UVP; Upland, CA, USA).

### 4.9. EGFP-LC3 Fluorescence Microscopy

The cells were seeded in 24-well plates at (1 × 10^5^ cells/well) and transfected with 1 µg of the EGFP-LC3 plasmid each. The transfection media was replaced the following day with growth media only, or growth media containing 100 µM BBR, and then incubated further for 48 h. The cells were then fixed with 4% paraformaldehyde for 30 min, washed with PBS, and then stained with the Hoechst nuclear stain for imaging using a fluorescence microscope.

### 4.10. Statistical Analysis

GraphPad Prism software (Version 7.03, GraphPad Software) was used for the statistical analysis. The values represent mean ± standard deviation (SD). Student’s t-test was used for comparison and a *p* value of < 0.05 was considered to be statistically significant.

## Supplementary Materials

The following are available online at https://www.mdpi.com/2073-4409/9/4/908/s1, Figure S1: Co-treatment of non-cytotoxic concentrations of BBR and BAF induced hepatoma cell death similar to 100 μM of BBR alone, Figure S2: BBR treatment did not trigger PARP cleavage, Figure S3: BBR treatment marginally blocked HCV replication.
