# Supplementary material for: Targeting Autophagy Augments Berberine-Mediated Cell Death in Human Hepatoma Cells Harboring Hepatitis C Virus RNA
Source: Cells. 2020 Apr 8;9(4):908. doi: 10.3390/cells9040908 (PMC7226840; doi:10.3390/cells9040908)
Supplement: Supplementary file 1 [file cells-09-00908-s001.pdf]

Targeting Autophagy Augments BBR-Mediated Cell Death in Human Hepatoma Cells Harboring Hepatitis C Virus RNA

Supplementary Materials

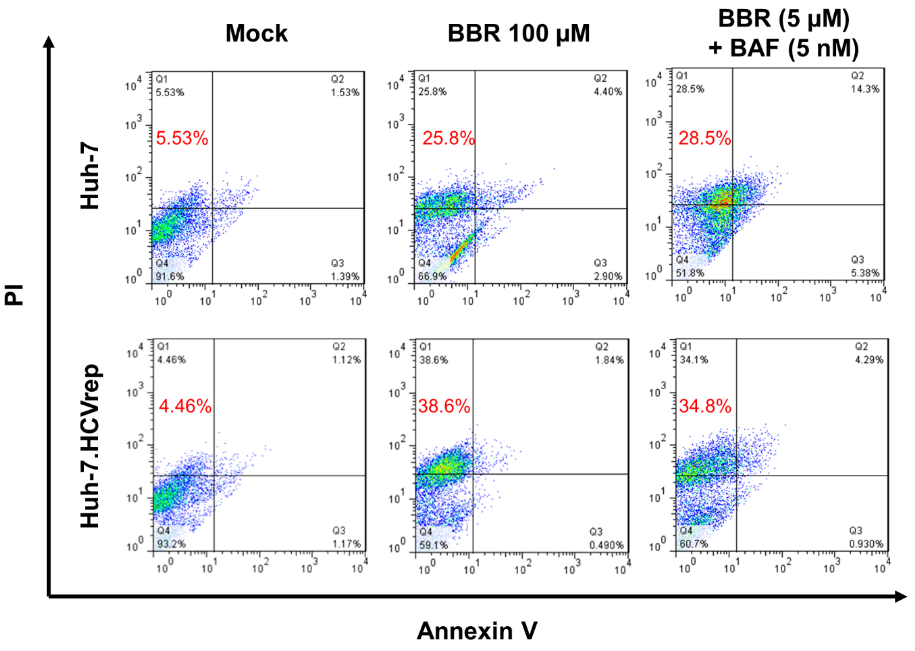

Figure S1. Co-treatment of non-cytotoxic concentrations of BBR and BAF induced hepatoma cell death similar to 100 μM of BBR alone. Cells were seeded in 6-well plates and co-treated with non-cytotoxic concentrations of BBR (5 μM) and BAF (5 nM) for 48 h. Cells were then trypsinized, stained with Annexin V and propidium iodide (PI), and analyzed by flow cytometry as indicated in the manuscript.

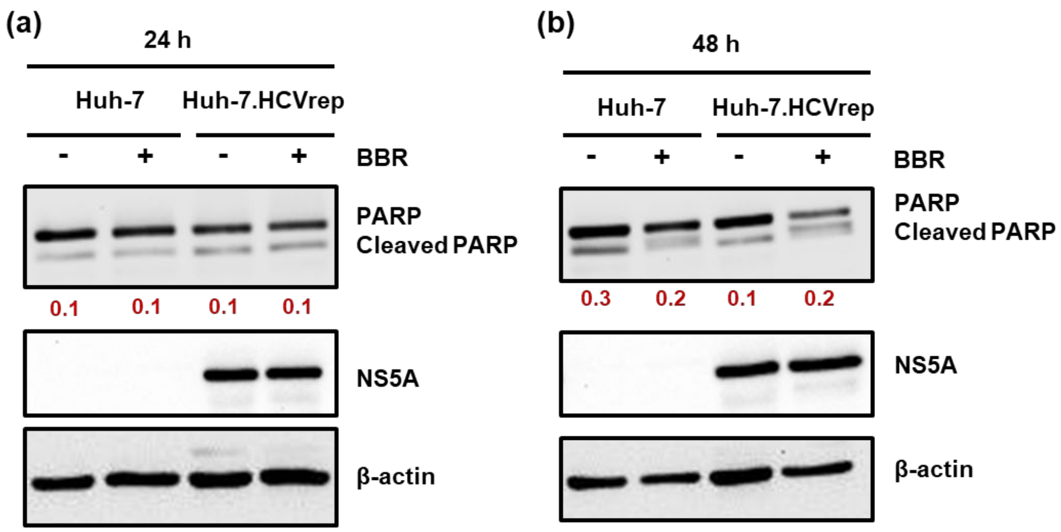

Figure S2. BBR treatment did not trigger PARP cleavage. The Huh-7 cells and the Huh-7.HCVrep cells were seeded in 6-well plates and treated with or without 100 μM BBR for (**a**) 24 h and (**b**) 48 h. The lysates were subsequently collected for a Western blot analysis against the indicated proteins.

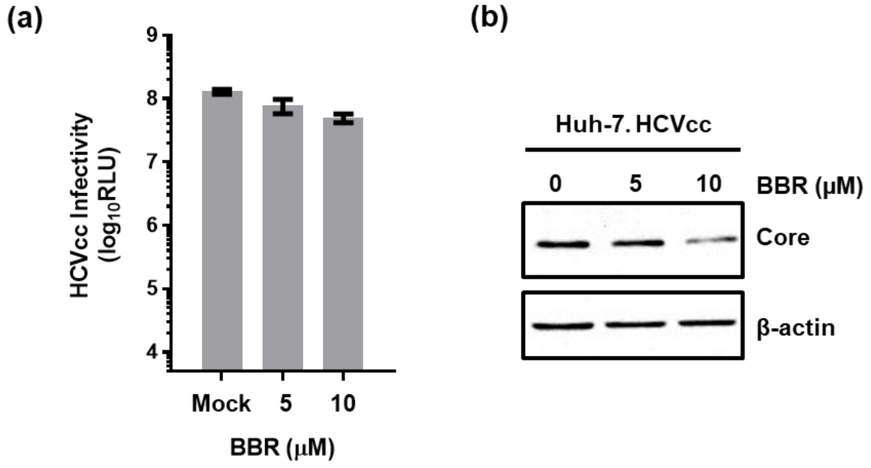

Figure S3. BBR treatment marginally blocked HCV replication. (**a**) The Huh-7.HCVcc cells were seeded in a 96-well plate and treated with the indicated non-cytotoxic concentrations of BBR for 72 h. The supernatants were then collected and assayed for the *Gaussia* luciferase activity. (**b**) Huh-7.HCVcc cells were seeded in 6-well plates and treated with or without BBR for 72 h. The lysates were subsequently collected for a Western blot analysis against the indicated proteins.
